# Trends of Testicular Cancer Mortality-to-Incidence Ratios in Relation to Health Expenditure: An Ecological Study of 54 Countries

**DOI:** 10.3390/ijerph18041546

**Published:** 2021-02-06

**Authors:** Shao-Chuan Wang, Nai-Wen Chang, Wen-Jung Chen, Min-Hsin Yang, Sung-Lang Chen, Wen-Wei Sung

**Affiliations:** 1 Department of Urology, Chung Shan Medical University Hospital, Taichung 40201, Taiwan; rosenbeck.wang@gmail.com (S.-C.W.); catastream@gmail.com (N.-W.C.); mimic1024@gmail.com (W.-J.C.); barbarian06070136@gmail.com (M.-H.Y.); 2 School of Medicine, Chung Shan Medical University, Taichung 40201, Taiwan; 3 Institute of Medicine, Chung Shan Medical University, Taichung 40201, Taiwan

**Keywords:** testicular neoplasms, mortality, incidence, health expenditures

## Abstract

Favorable testicular cancer mortality-to-incidence ratios (MIRs) are associated with health care disparities, including health care expenditures, but the trends of testicular MIR and health care disparity remain unclear. We evaluated changes in MIR as the difference between 2012 and 2018, termed delta MIR (*δ*MIR). Health care expenditures and the human development index (HDI) were obtained from the World Health Organization and the Human Development Report Office of the United Nations Development Programme. The association between the variables was analyzed by Spearman’s rank correlation coefficient. A total of 54 countries were included in the criteria of data quality reports and missing data. By continent, the most favorable MIR was in Oceania (0.03) while it was 0.36 in Africa. In these areas, the incidence rates were positively correlated to health care expenditure, but the mortality rates showed a reversed correlation. The MIR ranged from 0.01 to 0.34 and the *δ*MIR ranged from −0.05 to 0.34. The favorable MIRs are correlated to high health care expenditure and HDI (all *p* < 0.001). Interestingly, favorable *δ*MIRs tend to be seen in countries with relatively low health care expenditure and HDI (all *p* < 0.001). In conclusion, favorable testicular cancer MIRs are associated with high HDI and health care expenditure, but the improvement in MIR between 2012 and 2018 (*δ*MIR) is negatively correlated with HDI and health care expenditure.

## 1. Introduction

Although testicular cancer is a relatively rare neoplasm, accounting for just under 1% of cancers and ranking 26th for cancer incidence in men, the annual incidence increased 1.80-fold over 25 years, from 37,231 in 1990 to 66,833 new cases in 2016, with the increase particularly notable in Caucasian males [1,2]. Over the decades, the gap between high- and low-incidence countries has narrowed, mainly within Europe, with the incidence stabilizing in high-incidence countries while increasing in some formerly low-incidence countries [3]. This trend may be related to the improved detection of testicular cancer and the qualities of the cancer registries in the formerly low-incidence countries [4]. Additionally, it seems to be associated with increased exposure to environmental factors, such as maternal exposure to exogenous toxins and chemical pollutants [5,6]. Known risk factors have been established, such as cryptorchidism, a previous diagnosis of testicular cancer, maternal estrogen exposure, family history, and ethnicity. Although the multifactorial etiology remains unclear, the risk factors described above have led to geographical variations in the distribution of the incidence of testicular cancer [7].

When we considered various geographical areas, the incidence rate varied considerably: Western and Northern Europe had the highest incidence, and the lowest rates were observed in Asia and Africa [1,8]. Nevertheless, the mortality rate was relatively low in Western and Northern Europe, suggesting the beneficial effects of a prompt diagnosis followed by effective multimodal treatment and surveillance [8]. Conversely, despite the incidence being low in Africa and Asia, their high mortality rate demonstrates a lack of effective detection and access to health care and treatment. Moreover, there is a positive correlation between the human development index (HDI, an index of average achievement in key dimensions of human development) and the standardized incidence rate of testicular cancer and a negative correlation with the standardized mortality rate; testicular cancer’s incidence increases with rising gross domestic product (GDP) per capita [9,10,11].

Among the epidemiological models, mortality-to-incidence ratio (MIR) is a feasible method to illustrate the global trends although it cannot replace the role of a cohort survey [12,13,14,15,16,17,18]. Our previous study showed that a favorable MIR for testicular cancer was associated with a better World Health Organization ranking and a higher total expenditure on health/GDP [19]. However, updated data enabled an analysis of MIR trends and an investigation of the association between the disparities of countries and the improvement in MIR. This study describes the trends for this disease by clarifying the associations between the HDI, current health expenditure (CHE), testicular cancer’s incidence and mortality, and the change in MIR, termed delta MIR (δMIR) during the period of 2012–2018.

## 2. Materials and Methods

**Figure 1:**
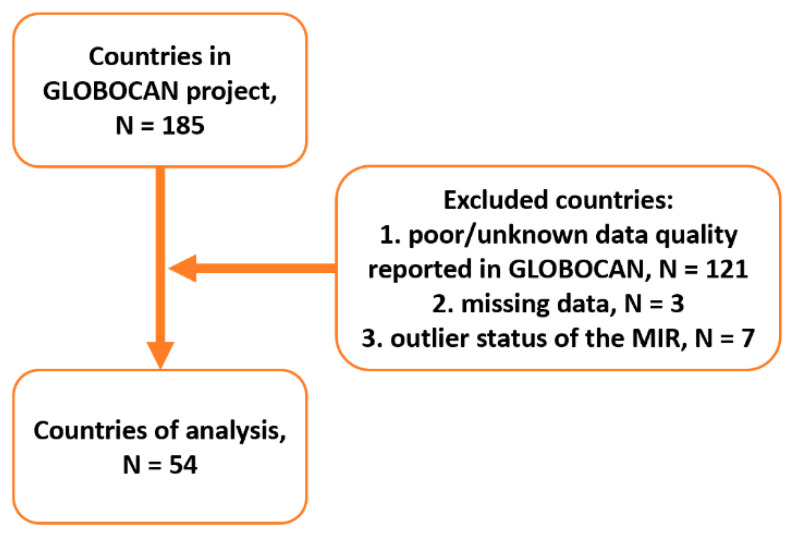
Diagram for data source selection.

This ecological study included databases from World Health Organization and Human Development Report Office. Cancer epidemiological data were obtained from the GLOBOCAN project maintained by the International Agency for Research on Cancer, World Health Organization (http://gco.iarc.fr/, accessed on 16 May 2020). The GLOBOCAN project is a public access database that provides contemporary estimates of cancer epidemiology for 185 countries. The exclusion criteria for country selection were based on the data quality report of GLOBOCAN (N = 121), missing data (N = 3), and the outlier status of the MIR (N = 7) (Figure 1). Countries with data quality reports of GLOBOCAN of high-quality national/regional data and complete vital registration were included. A total of 54 countries were included in the final analysis. The HDI score which is a summary measure of average achievement in key dimensions of human development including a long and healthy life, being knowledgeable, and having a decent standard of living, was obtained from the United Nations Development Programme, Human Development Report Office (http://hdr.undp.org/en, accessed on 16 May 2020). The health expenditure data, including CHE per capita and ratio of CHE to GDP by percentage (CHE/GDP), were obtained from the World Health Statistics (https://www.who.int/gho/publications/world_health_statistics/en/, accessed on 16 May 2020). The MIR is defined as the ratio of the crude rate (CR) of mortality and the CR of incidence as previously described [13,16,18,20]. *δ*MIR is defined as the difference between the MIR in 2012 and that in 2018 (*δ*MIR = MIR [in 2012] − MIR [in 2018]). The associations between the testicular cancer MIR, *δ*MIR, and factors among various countries were estimated by Spearman’s rank correlation coefficient using SPSS statistical software version 15.0 (SPSS Inc., Chicago, IL, USA). *p* values of < 0.05 were considered statistically significant. The scatterplot was generated via Microsoft Excel.

## 3. Results

### 3.1. Incidence, Mortality, and MIR of Testicular Cancer in Five Continents

To understand the epidemiology of testicular cancer in five continents, we calculated the number, incidence, mortality, and MIR of testicular cancer based on region (Table 1). Among all continents, Europe had the highest age-standardized rate (ASR) for incidence (6.3) and the second-highest ASR for mortality (0.35). The MIR in Europe was 0.07, a relatively low level compared to other continents. Although Africa had the lowest ASR for incidence and mortality (0.34 and 0.16, respectively), the MIR in Africa was the highest among the five continents (0.36). The other continents—Latin America and the Caribbean, North America, and Oceania—had a much higher ASR for incidence than Africa and Asia (4.7, 5.3, and 5.7 vs. 0.34 and 0.77, respectively). Nevertheless, there was no obvious difference in the ASR for mortality but rather a close interval between all the continents (ASR mortality: 0.16–0.54). The cumulative risk reflected the same ranking sequence as the ASR for incidence and, like the ASR for mortality, a close interval. The highest risk of incidence was in Europe and the lowest in Africa. However, there was little difference between the regions in the risk of mortality.

### 3.2. A Country’s HDI and CHE Are Significantly Associated with a Favorable MIR for Testicular Cancer

To assess the association between epidemiologic differences among countries and the disparities of country development as well as health care expenditure, we analyzed the countries based on the HDI, CHE per capita, and CHE/GDP, which are shown in Table 2. Among the selected countries, Norway had the highest HDI ranking, Switzerland had the highest CHE per capita (9818), and the United States of America had the highest CHE/GDP (16.8). As for the ASR for incidence and mortality, the highest incidence was in Croatia (12.50), and the highest mortality was in Chile (1.10). Regarding the MIR of individual countries in 2018, South Africa, Belarus, Egypt, and Thailand had a higher ratio than other countries (0.34, 0.24, 0.19, and 0.19, respectively) (Table 2).

**Figure 2:**
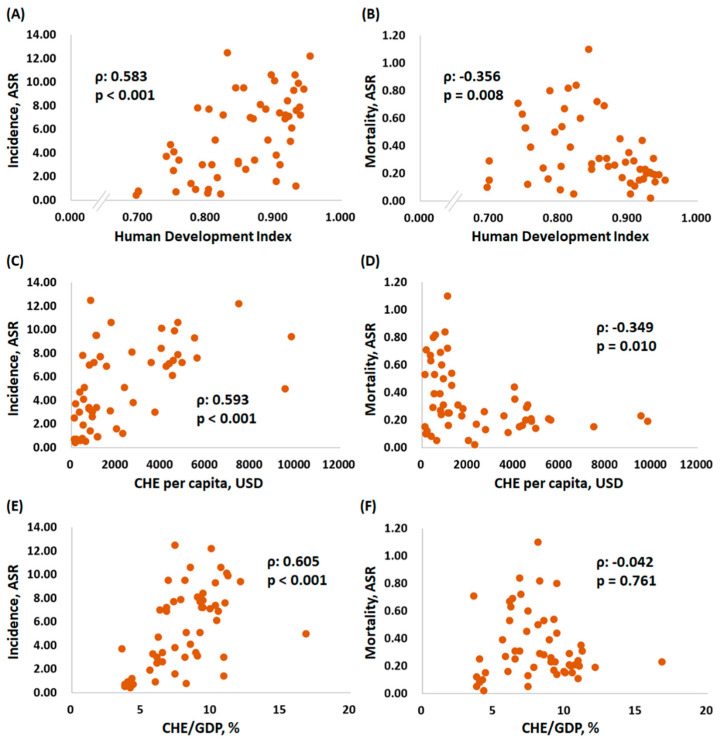
The association between incidence and mortality and human development index (HDI) (**A**,**B**), current health expenditure (CHE) per capita (**C**,**D**), and CHE/gross domestic product (CHE/GDP) (**E**,**F**) in testicular cancer.

**Figure 3:**
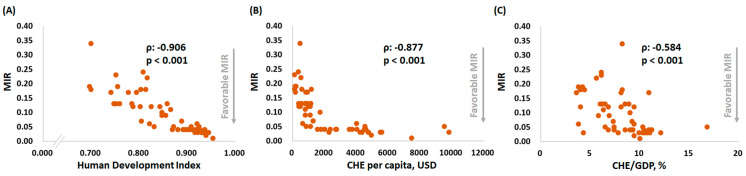
The (**A**) HDI, (**B**) CHE per capita, and (**C**) CHE as a percentage of GDP are significantly associated with the mortality-to-incidence ratios (MIR) in testicular cancer.

We further correlated the HDI, CHE per capita, and CHE/GDP with the ASR and MIR of testicular cancer by country (Figure 2 and Figure 3). Countries with a higher HDI, CHE per capita, or CHE/GDP had a higher ASR for incidence (ρ = 0.583, *p* < 0.001; ρ = 0.593, *p* < 0.001; and ρ = 0.605, *p* < 0.001, respectively) (Figure 2A,C,E). Countries with a higher HDI or CHE per capita had a higher ASR for mortality (ρ = −0.356, *p* = 0.008 and ρ = −0.349, *p* = 0.010, respectively) (Figure 2B,D). Nevertheless, no statistically significant difference for the ASR of mortality was found among the countries with various CHE/GDPs (ρ = −0.042, *p* = 0.761) (Figure 2F). Regarding the MIR, a higher HDI, a higher CHE per capita, and a higher CHE/GDP were associated with a favorable MIR (ρ = −0.906, *p* < 0.001; ρ = −0.877, *p* < 0.001; and ρ = −0.584, *p* < 0.001, respectively) (Figure 3), illustrating that a country’s HDI ranking, CHE per capita, and CHE/GDP are significantly associated with a favorable MIR for testicular cancer.

### 3.3. A Country’s HDI and CHE Are Significantly Associated with an Unfavorable *δ*MIR for Testicular Cancer

**Figure 4:**
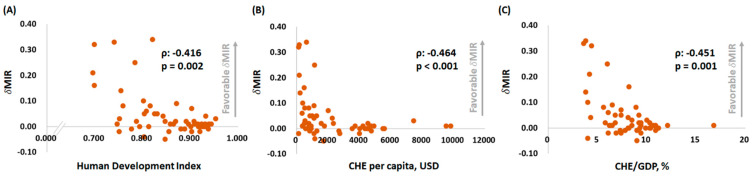
The (**A**) HDI, (**B**) CHE per capita, and (**C**) CHE as a percentage of GDP are significantly associated with the δMIR in testicular cancer.

To assess the trends of MIR between 2012 and 2018, we used the δMIR to identify disparities between the selected countries (Table 2). Among those countries, Fiji, Oman, and the Philippines had a δMIR of greater than 0.30, and there were 29 countries with a δMIR of less than 0.02 (Table 2). Surprisingly, concerning the association between δMIR and country disparities, a low HDI, low CHE per capita, and low CHE/GDP were associated with a favorable δMIR (ρ = −0.416, *p* = 0.002; ρ = −0.464, *p* < 0.001; and ρ = −0.451, *p* = 0.001, respectively) (Figure 4).

## 4. Discussion.

According to the GLOBOCAN estimates in 2012, about 55,300 new cases and 10,400 deaths occurred globally, and the GLOBOCAN estimates in 2018 show increasing annual new cases, with 71,000 diagnosed and 9500 deaths [21,22]. As to predicting the future, a European study suggests that the incidence will increase in 21 of 28 European countries from 2010 to 2035 [23]. As population aging continues, the incidence will increase despite the declining demographic impact of men aged 15–40, the population group that most commonly experiences testicular cancer [23,24]. Although the contributing drivers of this increase have not been fully described, a recent study suggests that environmental factors and lifestyle changes probably play important roles [2]. Due to the increasing burden of incidence, it is necessary to establish diagnosis by raising individual awareness. A review study found that some interventions, such as a television show, a university campaign, and interactive educational sessions, were successful in increasing men’s awareness and self-examination [25]. For successful treatment and long-term survival, early diagnosis facilitated by public awareness shifts the stage at presentation to that of a more localized and treatable disease.

The mortality rate of testicular cancer has attenuated globally over the past decades. Like previous studies, however, the present study found that many developing countries, especially in Latin America, have recently seen increases in mortality due to failing to cope with the increasing incidence [2,26]. Additionally, late presentation, poverty, and a lack of medical resources leading to uncompleted treatment courses are major challenges in developing countries. Studies in Nigeria and Tanzania found that an astonishing 33% and 39.3% of patients, respectively, presented with stage 4 disease [27,28]. Although all the patients received radical orchiectomy, only 25% received adjuvant radiotherapy without retroperitoneal lymph node dissection [27]. Reducing mortality in developing countries will require improvements in health care infrastructure, accessibility to health care, and health education to address the potential inequalities in the availability of health services.

Our previous study showed that a favorable MIR for testicular cancer was associated with higher total expenditure on health/GDP [19]. The results response to a previous study which also revealed a strong inverse relationship between HDI and MIR for testicular cancer [29]. HDI including education, life expectancy, and gross national income express a complex profile of social development than CHE per capita or CHE/GDP [9]. Through effective detection, optimal access to the healthcare system, and curative treatment, developed countries in Europe and North America, characteristic of high HDI, CHE, and CHE/GDP, have continued to maintain favorable MIRs and thus low δMIR. As for developing countries in Africa, the initial high MIRs demonstrate the necessity to make more efforts to improve the MIRs. Once they have dedicated to the establishment and the accessibility of the healthcare system, our data showed a corresponding improvement in the high δMIR.

There are some limitations to our study. Since the ecological study investigated collected databases, the results were prone to bias and confounding. We excluded countries with either poor data quality or little data to avoid misleading MIRs. However, this may have led to incompleteness in the data collection and a reduction in the generalizability of the results. In addition, the GLOBOCAN data may not accurately reflect the true incidence and mortality because of discrepancies in cancer registration between countries. Furthermore, mortality data are generally more accurate than incidence data in developing countries, which probably leads to the production of misleading MIRs. Moreover, the lack of information on comorbidities, ethnicity, risk factors (such as cryptorchidism), time to diagnosis, treatment, and annual data between the study period makes it difficult to understand how development is affecting cancer incidence and mortality. It is impossible to draw causal inferences about the effect of the HDI and CHE on testicular cancer as both the HDI and CHE are indicators of health care infrastructure for cancer care. Therefore, the MIR/δMIR cannot be directly attributed to either the HDI or CHE.

Despite these limitations, our study is the first to identify a correlation between HDI, CHE, and the δMIR for testicular cancer. The correlations were also confirmed with linear regression with all significant *p* values. Based on our previous study, we introduce the δMIR to reflect the trend of the disease. Countries with a low HDI or CHE per capita have a favorable MIR, indicating the improvement of these countries in testicular prognosis, especially in survival rates. Although large disparities in access to health care and oncological treatment still exist between developed and developing countries, we anticipate improvement through early diagnosis, the availability of multimodal treatment, elevated infrastructure care, and the identification of late relapse and salvage patients [29]. Although the MIR/δMIR are indicators for cancer prognosis, the MIR can inform health policy makers on areas of future research and health care investment, and the δMIR can indicate the quality and accessibility trends of a health care system over an interval.

## 5. Conclusions

The high quality of testicular cancer care of the health care system in an individual country is associated with a lower MIR. We selected an interval of time, the δMIR, as an innovative, effective trending indicator that demonstrated favorable trends in countries with a low CHE and HDI.

## Figures and Tables

**Table 1:** Summary of the number, rank, and percentage of testicular cancer in all cancers according to the continent.

Region	Incidence				Mortality				MIR
	Number	CR	ASR	Cum. Risk	Number	CR	ASR	Cum. Risk	
Continent									
Africa	1784	0.28	0.34	0.03	666	0.1	0.16	0.02	0.36
Asia	19,178	0.83	0.77	0.06	4520	0.2	0.18	0.02	0.24
Europe	23,864	6.7	6.3	0.48	1571	0.44	0.35	0.03	0.07
Latin America and the Caribbean	15,200	4.7	4.4	0.33	1917	0.6	0.54	0.04	0.13
North America	9422	5.3	5.1	0.39	451	0.25	0.22	0.02	0.05
Oceania	1167	5.7	5.4	0.42	39	0.19	0.17	0.01	0.03

CR: crude rate; ASR: age-standardized rate; Cum.: cumulative; MIR: mortality-to-incidence ratio.

**Table 2:** Summary of human development index, current health expenditure, cancer incidence, cancer mortality, and mortality-to-incidence ratio in testicular cancer of selected countries.

Country	Human Development Index		Current Health Expenditure		Incidence			Mortality			Mortality-to-Incidence Ratio		
	Score	Rank	Per Capita	% of GDP	ASR	CR	Cum. Risk	ASR	CR	Cum. Risk	2012	2018	*δ*MIR
Argentina	0.825	47	998	6.8	7.20	7.80	0.55	0.84	0.93	0.07	0.17	0.12	0.05
Australia	0.939	3	4934	9.4	7.20	7.60	0.55	0.14	0.17	0.01	0.03	0.02	0.01
Austria	0.908	20	4536	10.3	7.40	8.10	0.58	0.29	0.40	0.03	0.07	0.05	0.02
Belarus	0.808	53	352	6.1	3.00	3.40	0.23	0.67	0.82	0.06	0.30	0.24	0.06
Belgium	0.916	17	4228	10.5	6.90	6.90	0.52	0.15	0.18	0.01	0.04	0.03	0.01
Brazil	0.759	79	780	8.9	3.40	3.60	0.25	0.39	0.45	0.03	0.21	0.13	0.08
Bulgaria	0.813	51	572	8.2	5.10	5.50	0.40	0.82	1.00	0.07	0.18	0.18	0.00
Canada	0.926	12	4508	10.4	6.10	6.30	0.46	0.20	0.25	0.02	0.04	0.04	0.00
Chile	0.843	44	1102	8.1	9.50	10.70	0.72	1.10	1.30	0.09	0.16	0.12	0.04
Colombia	0.747	90	374	6.2	4.70	5.30	0.36	0.63	0.71	0.05	0.14	0.13	0.01
Costa Rica	0.794	63	929	8.1	3.00	3.30	0.22	0.50	0.57	0.04	0.17	0.17	0.00
Croatia	0.831	46	852	7.4	12.50	13.60	0.96	0.60	0.71	0.05	0.10	0.05	0.05
Cuba	0.777	73	826	10.9	1.40	1.90	0.13	0.24	0.32	0.02	0.16	0.17	−0.01
Cyprus	0.869	32	1563	6.8	6.90	8.00	0.51	0.31	0.34	0.02	0.06	0.04	0.02
Czechia	0.888	27	1284	7.3	7.70	8.50	0.59	0.45	0.60	0.04	0.06	0.07	−0.01
Denmark	0.929	11	5497	10.3	9.30	9.50	0.72	0.21	0.25	0.02	0.03	0.03	0.00
Ecuador	0.752	86	530	8.5	4.10	4.40	0.30	0.53	0.57	0.04	0.16	0.13	0.03
Egypt	0.696	115	157	4.2	0.43	0.43	0.04	0.10	0.08	0.01	0.40	0.19	0.21
Estonia	0.871	30	1112	6.5	3.40	3.50	0.25	0.25	0.17	0.02	0.14	0.05	0.09
Fiji	0.741	92	175	3.6	3.70	3.90	0.28	0.71	0.65	0.08	0.50	0.17	0.33
Finland	0.920	15	4005	9.4	8.40	8.20	0.62	0.44	0.48	0.04	0.04	0.06	−0.02
France	0.901	24	4026	11.1	10.10	10.20	0.78	0.35	0.40	0.03	0.04	0.04	0.00
Germany	0.936	5	4592	11.2	9.90	10.90	0.79	0.31	0.42	0.03	0.04	0.04	0.00
Ireland	0.938	4	4757	7.8	7.90	8.10	0.60	0.19	0.21	0.02	0.02	0.03	−0.01
Israel	0.903	22	2756	7.4	3.80	3.80	0.29	0.13	0.14	0.01	0.02	0.04	−0.02
Italy	0.880	28	2700	9.0	8.10	7.70	0.60	0.26	0.31	0.02	0.03	0.04	−0.01
Japan	0.909	19	3733	10.9	3.00	3.20	0.24	0.11	0.13	0.01	0.05	0.04	0.01
Kuwait	0.803	56	1169	4.0	0.90	0.95	0.06	0.25	0.17	0.01	0.14	0.18	−0.04
Latvia	0.847	41	784	5.8	3.30	4.00	0.28	0.27	0.34	0.02	0.11	0.09	0.02
Lithuania	0.858	35	923	6.5	2.60	3.00	0.21	0.31	0.38	0.03	0.14	0.13	0.01
Malaysia	0.802	57	386	4.0	0.59	0.66	0.04	0.08	0.08	0.01	0.22	0.12	0.10
Netherlands	0.931	10	4746	10.7	10.60	10.30	0.79	0.21	0.26	0.02	0.04	0.03	0.01
New Zealand	0.917	16	3554	9.3	7.20	7.40	0.56	0.23	0.26	0.02	0.04	0.04	0.00
Norway	0.953	1	7464	10.0	12.20	13.00	0.94	0.15	0.19	0.01	0.04	0.01	0.03
Oman	0.821	48	636	3.8	0.52	1.00	0.03	0.05	0.06	0.00	0.40	0.06	0.34
Philippines	0.699	113	127	4.4	0.69	0.66	0.06	0.15	0.12	0.02	0.50	0.18	0.32
Poland	0.865	33	797	6.3	7.00	7.70	0.53	0.69	0.87	0.06	0.12	0.11	0.01
Portugal	0.847	41	1722	9.0	3.10	3.00	0.22	0.23	0.31	0.02	0.05	0.10	−0.05
Russian Federation	0.816	49	524	5.6	1.90	2.20	0.15	0.39	0.49	0.03	0.30	0.22	0.08
Serbia	0.787	67	491	9.4	7.80	8.00	0.57	0.80	0.99	0.07	0.14	0.12	0.02
Singapore	0.932	9	2280	4.3	1.20	1.30	0.09	0.02	0.04	0.00	0.07	0.03	0.04
Slovakia	0.855	38	1108	6.9	9.50	10.10	0.69	0.72	0.88	0.06	0.07	0.09	−0.02
Slovenia	0.896	25	1772	8.5	10.60	11.00	0.79	0.28	0.49	0.03	0.05	0.04	0.01
South Africa	0.699	113	471	8.2	0.76	0.77	0.07	0.29	0.26	0.03	0.50	0.34	0.16
South Korea	0.903	22	2013	7.4	1.60	1.50	0.11	0.05	0.06	0.00	0.11	0.04	0.07
Spain	0.891	26	2354	9.2	5.10	5.00	0.37	0.17	0.21	0.01	0.06	0.04	0.02
Sweden	0.933	7	5600	11.0	7.60	7.80	0.58	0.20	0.24	0.02	0.03	0.03	0.00
Switzerland	0.944	2	9818	12.1	9.40	10.30	0.74	0.19	0.26	0.02	0.04	0.03	0.01
Thailand	0.755	83	217	3.8	0.70	0.74	0.05	0.12	0.14	0.01	0.33	0.19	0.14
Trinidad and Tobago	0.784	69	1146	6.0	0.91	1.20	0.08	0.16	0.15	0.01	0.38	0.13	0.25
Ukraine	0.751	88	125	6.1	2.50	2.90	0.20	0.53	0.67	0.05	0.21	0.23	−0.02
United Kingdom	0.922	14	4356	9.9	7.10	7.60	0.57	0.16	0.20	0.01	0.03	0.03	0.00
United States of America	0.924	13	9536	16.8	5.00	5.20	0.38	0.23	0.25	0.02	0.06	0.05	0.01
Uruguay	0.804	55	1281	9.2	7.70	8.20	0.58	0.54	0.60	0.05	0.12	0.07	0.05

## Data Availability

There was no additional unpublished data.
